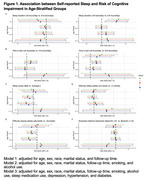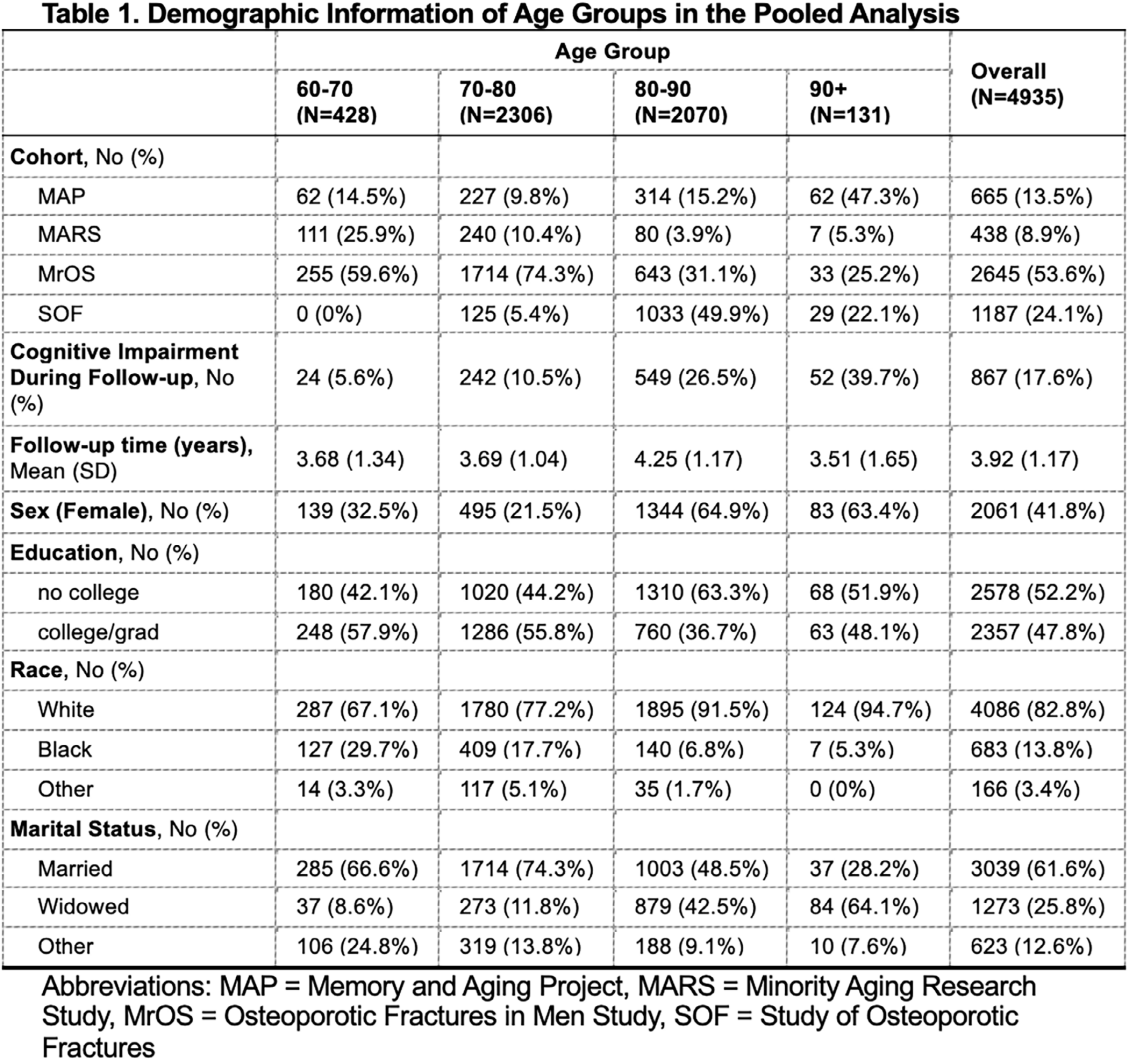# Subjective Sleep Characteristics and Cognitive Impairment from Ages 60 to 90+: A Pooled Analysis of Four Prospective Cohorts

**DOI:** 10.1002/alz70857_103902

**Published:** 2025-12-25

**Authors:** Yi Fang, Allysa Quick, Sasha Milton, Kristine Yaffe, Katie L Stone, Andrew Lim, Lisa L. Barnes, David A. A. Bennett, Meredith L. Wallace, Yue Leng

**Affiliations:** ^1^ University of California, San Francisco, San Francisco, CA, USA; ^2^ University of Pittsburgh, Pittsburgh, PA, USA; ^3^ California Pacific Medical Center Research Institute, San Francisco, CA, USA; ^4^ University of Toronto, Toronto, ON, Canada; ^5^ Rush Alzheimer's Disease Center, Rush University Medical Center, Chicago, IL, USA; ^6^ Rush University Medical Center, Chicago, IL, USA

## Abstract

**Background:**

Aging is associated with changes in sleep and cognitive function. While disrupted sleep is linked to cognitive decline, little is known about how subjective sleep characteristics relate to cognitive impairment across different age groups, particularly in the oldest old.

**Method:**

We harmonized data on self‐reported sleep, cognitive outcomes, and covariates across four U.S. cohorts: the Memory and Aging Project (MAP), Minority Aging Research Study (MARS), Osteoporotic Fractures in Men Study (MrOS), and Study of Osteoporotic Fractures (SOF). Sleep measures included sleep duration, time in bed, subjective sleep quality, excessive daytime sleepiness, and difficulty falling/staying asleep. Cognitive outcomes, including mild cognitive impairment (MCI) and dementia, were assessed through clinical diagnoses, crosswalk MMSE scores (MrOS and SOF), and adjudicated diagnosis (MAP and MARS). Using Poisson regression, we examined associations between sleep characteristics and incident cognitive impairment in age‐stratified groups (60‐70, 70‐80, 80‐90, 90+ years), adjusting for age, sex, race, marital status, follow‐up time, smoking, alcohol use, sleep medication use, depression, hypertension, and diabetes.

**Result:**

After exclusion of baseline MCI and dementia (*N* = 208), the pooled cohort (*N* =  4935; 60‐70: *N* = 428, 70‐80: *N* = 2306, 80‐90: *N* = 2070, 90+: *N* = 131) included 2061 (41.8%) females and 849 (17.2%) non‐White participants. During a mean follow‐up of 3.92±1.17 years, 867 (17.6%) developed MCI or dementia. Prolonged sleep duration (>8 hours/day) in the 90+ age group (Risk Ratio [RR] = 2.167, 95% CI 1.327∼3.539, *p* = 0.002), and excessive time in bed (> 8 hours/day) in the 80‐90 age group (RR = 1.201, 95% CI 1.037∼1.389, *p* = 0.014) were associated with an increased risk of cognitive impairment. Difficulty staying asleep >=3 times /week was associated with lower risk of cognitive impairment in the 80‐90 age group (RR = 0.831, 95% CI 0.720∼0.959, *p* = 0.011). No significant associations were observed in other age groups and for other sleep characteristics.

**Conclusion:**

Prolonged sleep duration and excessive time in bed were associated with a higher risk of cognitive impairment in adults over 80, independent of comorbidities. These findings highlight somnolence as a potential marker for cognitive impairment in advanced aging, suggesting that tailored sleep monitoring could aid detection.